# Changes in psoas muscle area in patients with colorectal cancer three years after surgery: a longitudinal approach

**DOI:** 10.1007/s00423-026-04002-9

**Published:** 2026-03-12

**Authors:** Helin Yikilmaz Pardes, Mona Sadek Ali, Troels Gammeltoft Dolin, Casper Simonsen, Lykke Sylow, Jakob Lykke, Jacob Rosenberg, Tinne Laurberg, Louise Lang Lehrskov

**Affiliations:** 1 Department of Surgery, Zealand University Hospital, University of Copenhagen, Lykkebaekvej 1, Køge, 4600 Denmark; 2 Department of Surgery, Copenhagen University Hospital – Herlev and Gentofte, Herlev, Denmark; 3 Department of Biomedical Sciences, Faculty of Health and Medical Sciences, University of Copenhagen, Copenhagen, Denmark; 4 Department of Medicine, Copenhagen University Hospital – Herlev and Gentofte, Herlev, Denmark; 5 Department of Oncology, Copenhagen University Hospital – Herlev and Gentofte, Herlev, Denmark; 6 Department of Pathology, Aarhus University Hospital, Aarhus, Denmark; 7 Steno Diabetes Center Aarhus, Aarhus University Hospital, Aarhus, Denmark; 8 Centre for Physical Activity Research, Copenhagen University Hospital – Rigshospitalet, Copenhagen, Denmark

**Keywords:** Colorectal Cancer, Cancer surgery, Postoperative muscle loss, Psoas muscle area

## Abstract

**Purpose:**

The purpose of this study was to investigate long-term changes in psoas muscle area after colorectal cancer (CRC) surgery and their association with preoperative clinical characteristics and mortality. Skeletal muscle loss predicts poor CRC outcomes, but evidence on postoperative changes is limited.

**Methods:**

We included 272 patients with localized CRC who underwent elective surgery at Herlev Hospital, Denmark, between 2014 and 2019. Computed tomography (CT) scans before surgery and at three-year follow-up were used to assess psoas muscle area at the L3 vertebral level. Change in muscle area was categorized as > 5% decrease, no change, or > 5% increase. Associations between preoperative clinical characteristics and mortality were analyzed in relation to muscle change.

**Results:**

At three-year follow-up after surgery, 39% of patients showed a > 5% decrease in psoas muscle area, 33% had no change, and 28% had a > 5% increase. The results demonstrated that both Union for International Cancer Control (UICC) tumor stage and receipt of adjuvant chemotherapy were independently associated with postoperative changes in psoas muscle area. Among patients who did not receive adjuvant chemotherapy, age was associated with muscle area change, whereas no preoperative clinical characteristics were associated with muscle area change among those receiving adjuvant chemotherapy. Importantly, postoperative longitudinal changes in psoas muscle area were not associated with overall mortality.

**Conclusion:**

A considerable proportion of patients with CRC experience long-term loss of psoas muscle area following surgery. These results underscore the variability in postoperative muscle changes and highlight the need for further research to identify high-risk subgroups.

**Supplementary Information:**

The online version contains supplementary material available at 10.1007/s00423-026-04002-9.

## Introduction

Colorectal cancer (CRC) remains the third most common malignancy worldwide, with an increasing incidence particularly among patients over the age of 50 [1]. With the incidence being more than fifty times higher in patients aged 60–70 than in younger patients, there has been a significant emphasis on improving long-term surgical, oncological and survival outcomes among the elderly patients [2]. Despite substantial progress in perioperative care with the implementation of enhanced recovery after surgery protocols (ERAS^®^) and prehabilitation, the risk of postoperative complications, prolonged recovery, and increased mortality remain an important concern [3]. Identifying factors associated with adverse outcomes in patients undergoing surgery with a curative intent for CRC is therefore of critical importance.

Recent research suggest that loss of muscle mass could be an important factor in predicting long-term outcomes for patients with CRC [4]. In patients with cancer, the age-related progressive loss of both muscle mass and muscle strength is often accelerated, substantially increasing the risk of functional limitations, frailty, morbidity and impaired quality of life [5–10]. Systemic inflammation, cancer-induced metabolic alterations, and treatment-related factors, including chemotherapy, have been implicated in driving muscle depletion in patients with CRC [11]. Adjuvant chemotherapy has been notably associated with skeletal muscle loss, often termed chemotherapy-induced sarcopenia [12].

Current postoperative surveillance programs for patients with CRC do not include targeted interventions for significant muscle loss. Developing such interventions requires a better understanding of the underlying mechanisms and identifying the patient subgroups most vulnerable to muscle loss. If changes in psoas muscle area can be shown to predict adverse outcomes such as mortality, they could be incorporated into multidisciplinary team (MDT) discussions to improve risk stratification and guide more individualized follow-up strategies. However, only a few studies have investigated the long-term changes in skeletal muscle area in patients with CRC. This study aimed to investigate longitudinal postoperative changes in psoas muscle area—an established surrogate marker for whole-body skeletal muscle—and their associations with preoperative clinical characteristics and mortality over a three-year period following CRC surgery.

## Methods

### Study design and population

This cohort-study was based on patients from the clinical REBECCA study “Biomarkers in patients with colorectal cancer - can they provide new information of the disease, effects of treatment, adverse events and prognosis?”. A prospective cohort of 401 patients undergoing elective surgery for CRC with a curative intent between July 2014 and December 2019. All patients with histologically verified or suspected CRC stages I–III were consecutively included in the REBECCA database at the Department of Gastroenterology, Herlev and Gentofte Hospital, Denmark. The cohort was established to enable systematic and prospective collection of clinical, pathological, and biomarker data, with the overarching aim of investigating the prognostic and predictive significance of biomarkers in relation to disease course, treatment effects, adverse events, and outcomes in CRC [13]. All surgeries were performed by specialized colorectal surgeons. Following surgery, some of the patients were treated for 3–6 months with adjuvant chemotherapy depending on the Union for International Cancer Control (UICC) cancer stage, in accordance with Danish treatment guidelines issued by Danish Colorectal Cancer Group (DCCG) [14]. In brief, adjuvant chemotherapy was routinely recommended for patients with stage III disease after radical resection, while in stage II disease, it was administered selectively to individuals exhibiting high-risk features.

Patients were excluded from the current study if they had received neoadjuvant chemotherapy, had recurrent disease, or did not have a follow-up scan. Follow-up was defined as the period from the date of the three-year postoperative CT scan until death or censoring at the time of vital status ascertainment.

### Data sources

Data was extracted from electronic medical records. Preoperative characteristics, including demographic information (age, sex), lifestyle factors (body mass index (BMI), tobacco use, and alcohol consumption), American Society of Anesthesiologists (ASA) score, UICC cancer stage and treatment modalities (surgical and oncological) and survival, were retrieved and included in the REBECCA database.

C-reactive protein (CRP) concentrations were consecutively measured as part of the clinical blood work using high-sensitive CRP ultra-ready-to-use, liquid assay reagent via an immunoturbidimetric method on a fully automated chemistry analyzer (Kit-test SENTINEL CRP Ultra (UD), 11508 UD-2.0/02 2015/09/23) at the Department of Clinical Biochemistry.

### CT scans

CT scans were performed preoperatively for diagnostic purposes with follow-up imaging performed three years after surgery in accordance with the standard protocols of the Danish CRC surveillance program [14]. Consequently, it was not possible to include patients who died within three years after surgery.

Studies have validated the use of L3 as a reliable landmark for muscle measurement, as it strongly correlates with whole-body muscle mass [14, 15]. Pre- and postoperative measurements were independently performed by two investigators (HYP and MSA). Each measurement was conducted once, and inter-rater reliability was not evaluated.

When assessing the CT scans, the third lumbar (L3) vertebra (axial image where the coronal plane was visible) was selected as a standardized landmark on cross-sectional imaging. Each image was analyzed using the software *IMPAX Client*, which allows manual tracing of regions of interest. The psoas muscles on the right and left side were manually outlined, after which the software automatically calculated the muscle area in square centimeters (cm²). Hounsfield Units were used as a reference to ensure accurate measurement, helping to confirm that muscle, fat, and connective tissue were correctly identified.

The change in muscle area from the preoperative scans to the three-year follow-up was defined as: (muscle area at 3-years – muscle area at baseline)/muscle area at baseline. Relative changes in muscle area of less than 5% were considered as “no change,” while the changes were categorized as either a decrease of greater than 5% or an increase of greater than 5%. Subsequently, patients were classified into subgroups based on the magnitude of muscle area change, using the following categories: >20%, 10–20%, 5–10%, or 0–5% increase or decrease.

### Outcome measures

The primary objective of this study was to examine changes in muscle area over a three-year period in patients who underwent surgery for CRC and to investigate whether these changes were associated with preoperative clinical and pathological factors (e.g. preoperative CRP level, UICC cancer stage, type of surgical procedure and adjuvant chemotherapy) as well as mortality.

### Ethics statement

This study was conducted in accordance with the Declaration of Helsinki. All experimental protocols (The REBECCA study protocol (VEK j.nr. H-2–2013-078) and the additional protocol for the current project (78485) were approved by the Danish Ethical Committee of the Capital Region of Denmark and the Danish Data Protection Agency in Copenhagen, Denmark (HEH-2014-105; I-Suite 03330; PRIVACY P-2020-578). All patients received oral and written information about the study and gave their written consent before inclusion in accordance with the guidelines of the Danish Ethics Committee.

### Statistical analysis

Descriptive statistics were used to compare baseline characteristics across groups. Categorical variables were summarized as counts (n) and percentages (%). Continuous variables were presented as mean ± standard deviation (SD) or as median with interquartile range (25th–75th percentile) for non-normally distributed variables.

Differences in continuous variables were assessed using analysis of variance (ANOVA) or the Kruskal-Wallis rank test, as appropriate. Categorical variables were compared using Pearson’s chi-squared test or Fisher’s exact test in cases with small, expected counts.

A two-sided p-value < 0.05 was considered statistically significant. Kaplan-Meier survival analysis was used to assess all-cause mortality, and Cox proportional hazards regression analysis was performed to estimate mortality risk. All statistical analyses were conducted using Stata version 18.0 (StataCorp, College Station, TX, USA).

## Results

In total 401 patients with localized CRC who underwent surgical treatment were included in the REBECCA cohort. Of these, 129 patients were excluded due to absence of an available three-year follow-up CT scan. The final study population consisted of 272 patients, with an equal gender distribution. The psoas muscle area was smaller in females (12.5 cm², SD: 3.4) compared to males (18.5 cm², SD: 4.4) (Table 1). The mean BMI was slightly higher in men than in women (26.3 ± 3.7 vs. 25.4 ± 4.9 kg/m²); however, this difference did not reach statistical significance (*p* = 0.08). When BMI was categorized, a significant difference in BMI distribution between sexes was observed (*p* < 0.001). Underweight (BMI < 18.5 kg/m²) was observed only among women (3.5%), whereas a higher proportion of men were classified as overweight (BMI 25–<30 kg/m²) compared with women (50.0% vs. 28.9%). The prevalence of obesity (BMI ≥ 30 kg/m²) was comparable between men and women (22.3% vs. 23.9%). Apart from this, no sex-related differences were identified in the preoperative characteristics.

**Table 1:** Preoperative patient demographics at baseline

*n* (%)	Male	Female	Total	*P*-Value
	130 (47.8)	142 (52.2)	272 (100)	
Age, year, mean (sd)	67.3 (8.5)	66.1 (9.5)	66.7 (9.1)	0.26
BMI kg/m^2^, mean (sd)	26.3 (3.7)	25.4 (4.9)	25.8 (4.4)	0.08
BMI kg/m^2^, *n* (%)				
< 18.5	0	5 (3.5)	5 (1.8)	
≥ 18.5 and < 25	36 (27.7)	62 (43.7)	98 (36.0)	0.00
≥ 25 and < 30	65 (50.0)	41 (28.9)	106 (39.0)	
≥ 30	29 (22.3)	34 (23.9)	63 (23.2)	
Baseline mean of total psoas area, cm^2^ (sd)	18.5 (4.4)	12.5 (3.4)	15.4 (4.9)	0.00
CRP, mg/L, median (IQR)	3.0 (1.9–4.3)	3.0 (1.9–4.8)	3.0 (1.9–4.7)	0.81
Actively smoking, *n (*%)	18 (14.0)	18 (12.9)	36 (13.4)	0.79
Alcohol intake (> = 7 units pr week), *n* (%)	41 (32.0)	33 (23.7)	74 (27.7)	0.13
ASA score, *n* (%)				
1	48 (37.5)	66 (46.5)	114 (42.2)	
2	67 (52.3)	70 (49.3)	137 (50.7)	0.09
3	13 (10.2)	6 (4.2)	19 (7.0)	
UICC stage, *n* (%)				
I	49 (37.7)	52 (36.6)	101 (37.1)	
II	44 (33.8)	48 (33.8)	92 (33.8)	0.98
III	37 (28.5)	42 (29.6)	79 (29.0)	
Type of surgery, *n* (%)				
Hemicolectomy	41 (31.5)	64 (45.1)	105 (38.6)	
Sigmoidectomy	46 (35.4)	37 (26.1)	83 (30.5)	0.06
Rectum resection (Total mesorectal excision)	43 (33.1)	41 (28.9)	84 (30.9)	
Adjuvant chemotherapy, *n* (%)	39 (30.0)	52 (36.6)	91 (33.5)	0.25

Values are given as absolute numbers (percentages) in parentheses unless otherwise is stated

*BMI* Body Mass Index, *CRP* C-reactive protein, *ASA* American Society of Anesthesiology score, *UICC* Union for International Cancer Control

### Changes in psoas muscle area three years after surgery

At the three-year follow-up after CRC surgery, 106 patients (39%) experienced a reduction of more than 5% in psoas muscle area (Table 2). Patients with a >5% decrease, no change, or a >5% increase in psoas muscle area were comparable in terms of sex, age, BMI, smoking status, alcohol intake, ASA score, type of surgery, and mortality. Significant differences between groups were observed for baseline total psoas muscle area (p = 0.03), CRP levels (*p* = 0.01), and UICC stage distribution (*p* = 0.03). In addition, the proportion of patients receiving adjuvant chemotherapy differed significantly across groups (p < 0.001), with a higher prevalence among patients who experienced an increase in psoas muscle area.

**Table 2:** Preoperative baseline demographics and changes in psoas muscle area

*n* (%)	Decrease > 5%	No change	Increase > 5%	Total	*P*-value
	106 (39.0)	90 (33.1)	76 (27.9)	272 (100)	
Sex, *n* (%)					
Male	47 (44.3)	45 (50.0)	38 (50.0)	130 (47.8)	0.66
Female	59 (55.7)	45 (50.0)	38 (50.0)	142 (52.2)	
Age, year, mean (sd)	66.9 (8.7)	67.4 (8.2)	65.4 (10.4)	66.7 (9.1)	0.36
BMI kg/m^2^, mean (sd)	26.2 (4.4)	25.6 (5.2)	25.6 (3.5)	25.8 (4.4)	0.64
BMI kg/m^2^, *n* (%)					
< 18.5	1 (0.9)	4 (4.4)	0 (0.0)	5 (1.8)	
≥ 18.5 and < 25	34 (32.1)	36 (40.0)	28 (36.8)	98 (36.0)	0.25
≥ 25 and < 30	42 (39.6)	33 (36.7)	31 (40.8)	106 (39.0)	
≥ 30	29 (27.4)	17 (18.9)	17 (22.4)	63 (23.2)	
Baseline mean of total psoas area (sd)	16.2 (4.8)	15.4 (5.2)	14.2 (4.7)	15.4 (4.9)	0.03*
CRP, mg/L, median (IQR)	3.0 (1.3–4.0)	3.0 (1.9–4.0)	3.0 (3.0–7.5)	3.0 (1.9–4.7)	0.01*
Actively smoking, *n (*%)	13 (12.3)	12 (13.5)	11 (14.9)	36 (13.4)	0.88
Alcohol intake (> = 7 units pr week), *n* (%)	31 (29.5)	29 (33.3)	14 (18.7)	74 (27.7)	0.10
ASA score, *n* (%)					
1	44 (41.9)	39 (43.8)	31 (40.8)	114 (42.2)	
2	53 (50.5)	46 (51.7)	38 (50.0)	137 (50.7)	0.83
3	8 (7.6)	4 (4.5)	7 (9.2)	19 (7.0)	
UICC stage, *n* (%)					
I	51 (48.1)	29 (32.2)	21 (27.6)	101 (37.1)	
II	33 (31.1)	32 (35.6)	27 (35.5)	92 (33.8)	0.03*
III	22 (20.8)	29 (32.2)	28 (36.8)	79 (29.0)	
Type of surgery, *n* (%)					
Hemicolectomy	34 (32.1)	40 (44.4)	31 (40.8)	105 (38.6)	
Sigmoidectomy	33 (31.1)	24 (26.7)	26 (34.2)	83 (30.5)	0.29
Rectum resection (Total mesorectal excision)	39 (36.8)	26 (28.9)	26 (34.2)	84 (30.9)	
Adjuvant chemotherapy, *n* (%)	23 (21.7)	32 (35.6)	36 (47.4)	91 (33.5)	< 0.001*
Death, *n* (%)	8 (7.5)	10 (11.1)	5 (6.6)	23 (8.5)	0.53

Values are given as absolute numbers (percentages) in parentheses unless otherwise is stated

Statistical significance is denoted as follows: *p* < 0.05 (*)

*BMI* Body Mass Index, *CRP* C-reactive protein, *ASA* American Society of Anesthesiology score, *UICC* Union for International Cancer Control

Among the 272 patients, 32 (12%) had a decrease of over 20%, 46 (17%) had a decrease of 10-20%, and 28 (10%) had a decrease of 5-10% (Supplementary Table 1). Conversely, 80 patients (26%) had an increase in muscle area, with 22 (8.1%) showing an increase of over 20%, 27 (9.9%) having an increase of 10-20%, and 27 (9.9%) exhibiting an increase of 5-10%. Notably, patients with a loss of more than 20% in psoas muscle area were predominantly classified as overweight according to BMI; however, this association was not statistically significant (Supplementary Table 1).

### Stratified analysis based on treatment with adjuvant chemotherapy

Among patients who underwent surgery without receiving adjuvant chemotherapy (Table 3) 45.9% experienced a >5% decrease, 32.0% had no change, and 22.1% showed a >5% increase in psoas muscle area.

**Table 3:** Preoperative baseline demographics of patients who did not receive adjuvant chemotherapy and changes in psoas muscle area

*n* (%)	Decrease > 5%	No change	Increase > 5%	Total	*P*-value
	83 (45.9)	58 (32.0)	40 (22.1)	181 (100.0)	
Sex, *n* (%)					
Male	38 (45.8)	32 (55.2)	21 (52.5)	91 (50.3)	0.52
Female	45 (54.2)	26 (44.8)	19 (47.5)	90 (49.7)	
Age, year, mean (sd)	68.5 (7.8)	68.9 (8.1)	64.8 (11.0)	67.8 (8.8)	0.04*
BMI kg/m^2^, mean (sd)	25.8 (4.2)	25.6 (4.6)	26.0 (3.4)	25.8 (4.2)	0.88
BMI kg/m^2^, *n* (%)					
< 18.5	1 (1.2)	4 (6.9)	0 (0.0)	5 (2.8)	
≥ 18.5 and < 25	26 (31.3)	21 (36.2)	13 (32.5)	60 (33.1)	0.38
≥ 25 and < 30	36 (43.4)	22 (37.9)	18 (45.0)	76 (42.0)	
≥ 30	20 (24.1)	11 (19.0)	9 (22.5)	40 (22.1)	
Baseline mean of total psoas area (sd)	16.0 (4.6)	16.0 (5.3)	14.1 (4.3)	15.6 (4.8)	0.07
CRP, mg/L, median (IQR)	3.0 (1.3–4.0.3.0)	3.0 (2.0–3.4.0.4)	3.0 (3.0–6.9.0.9)	3.0 (1.9–4.7)	0.05
Actively smoking, *n* (%)	9 (10.8)	5 (8.8)	8 (20.0)	22 (12.2)	0.22
Alcohol intake (> = 7 units pr week), *n* (%)	24 (29.3)	21 (37.5)	7 (17.5)	52 (29.2)	0.10
ASA score, *n* (%)					
1	38 (46.3)	25 (43.9)	15 (37.5)	78 (43.6)	
2	40 (48.8)	30 (52.6)	19 (47.5)	89 (49.7)	0.20
3	4 (4.9)	2 (3.5)	6 (15.0)	12 (6.7)	
UICC stage, *n* (%)					
I	51 (61.4)	29 (50.0)	21 (52.5)	101 (55.8)	
II	29 (34.9)	26 (44.8)	16 (40.0)	71 (39.2)	0.63
III	3 (3.6)	3 (5.2)	3 (7.5)	9 (5.0)	
Type of surgery, *n* (%)					
Hemicolectomy	30 (36.1)	29 (50.0)	20 (50.0)	79 (43.6)	
Sigmoidectomy	25 (30.1)	11 (19.0)	13 (32.5)	49 (27.1)	0.16
Rectum resection (Total mesorectal excision)	28 (33.7)	18 (31.0)	7 (17.5)	53 (29.3)	
Death, *n* (%)	6 (7.2)	8 (13.8)	1 (2.5)	15 (8.3)	0.12

Values are given as absolute numbers (percentages) in parentheses unless otherwise is stated.

Statistical significance is denoted as follows: *p* < 0.05 (*)

*BMI* Body Mass Index, *CRP* C-reactive protein, *ASA* American Society of Anesthesiology score, *UICC* Union for International Cancer Control

The groups were largely comparable with respect to sex, BMI, smoking status, alcohol intake, ASA score, UICC stage, and type of surgery. Patients with an increase in psoas muscle area were slightly younger compared with the other groups (p = 0.04). No statistically significant differences were observed between groups for baseline psoas muscle area, CRP levels, or mortality.

In contrast, among patients who received adjuvant chemotherapy (Table 4), 25.3% experienced a >5% decrease, 35.2% had no change, and 39.6% showed a >5% increase in psoas muscle area. No statistically significant differences were observed across groups with respect to sex, age, BMI, smoking status, alcohol intake, ASA score, UICC stage, type of surgery, baseline psoas muscle area, or CRP levels. Mortality rates were also similar across groups during follow-up.

**Table 4:** Preoperative baseline demographics of patients who received adjuvant chemotherapy and changes in psoas muscle area

*n* (%)	Decrease > 5%	No change	Increase > 5%	Total	*P*-value
	23 (25.3)	32 (35.2)	36 (39.6)	91 (100.0)	
Sex, *n* (%)					
Male	9 (39.1)	13 (40.6)	17 (47.2)	39 (42.9)	0.79
Female	14 (60.9)	19 (59.4)	19 (52.8)	52 (57.1)	
Age, year, mean (sd)	60.9 (9.3)	64.8 (7.8)	66.2 (9.8)	64.4 (9.2)	0.09
BMI kg/m^2^, mean (sd)	27.3 (4.9)	25.7 (6.1)	25.2 (3.6)	25.9 (5.0)	0.31
BMI kg/m^2^ *n* (%)					
< 18.5	0 (0.0)	0 (0.0)	0 (0.0)	0 (0.0)	0.5
≥ 18.5 and < 25	8 (34.8)	15 (46.9)	15 (41.7)	38 (41.8)	
≥ 25 and < 30	6 (26.1)	11 (34.4)	13 (36.1)	30 (33.0)	
≥ 30	9 (39.6)	6 (18.8)	8 (22.2)	23 (25.3)	
Baseline mean of total psoas area (sd)	16.7 (5.4)	14.2 (4.7)	14.4 (5.2)	14.9 (5.1)	0.16
CRP, mg/L, median (IQR)	3.0 (1.2–4.6)	3.0 (1.9–6.1)	3.2 (2,7–10)	3.0 (1.9–4.7)	0.24
Actively smoking, *n (*%)	4 (17.4)	7 (21.9)	3 (8.8)	14 (15.7)	0.34
Alcohol intake (> = 7 units pr week), *n* (%)	7 (30.4)	8 (25.8)	7 (20.0)	22 (24.7)	0.66
ASA score, *n* (%)					
1	6 (26.1)	14 (43.8)	16 (44.4)	36 (39.6)	
2	13 (56.5)	16 (50.0)	19 (52.8)	48 (52.7)	0.24
3	4 (17.4)	2 (6.2)	1 (2.8)	7 (7.7)	
UICC stage, *n* (%)					
II	4 (17.4)	6 (18.8)	11 (30.6)	21 (23.1)	0.39
III	19 (82.6)	26 (81.2)	25 (69.4)	70 (76.9)	
Type of surgery, *n* (%)					
Hemicolectomy	4 (17.4)	11 (34.4)	11 (30.6)	26 (28.6)	
Sigmoidectomy	8 (34.8)	13 (40.6)	13 (36.1)	34 (37.4)	0.46
Rectum resection (Total mesorectal excision)	11 (47.8)	8 (25.0)	12 (33.3)	31 (34.1)	
Death, *n* (%)	2 (8.7)	2 (6.2)	4 (11.1)	8 (8.8)	0.78

Values are given as absolute numbers (percentages) in parentheses unless otherwise is stated

*BMI* Body Mass Index, *CRP* C-reactive protein, *ASA* American Society of Anesthesiology score, *UICC* Union for International Cancer Control

### Mortality

**Fig. 1:**
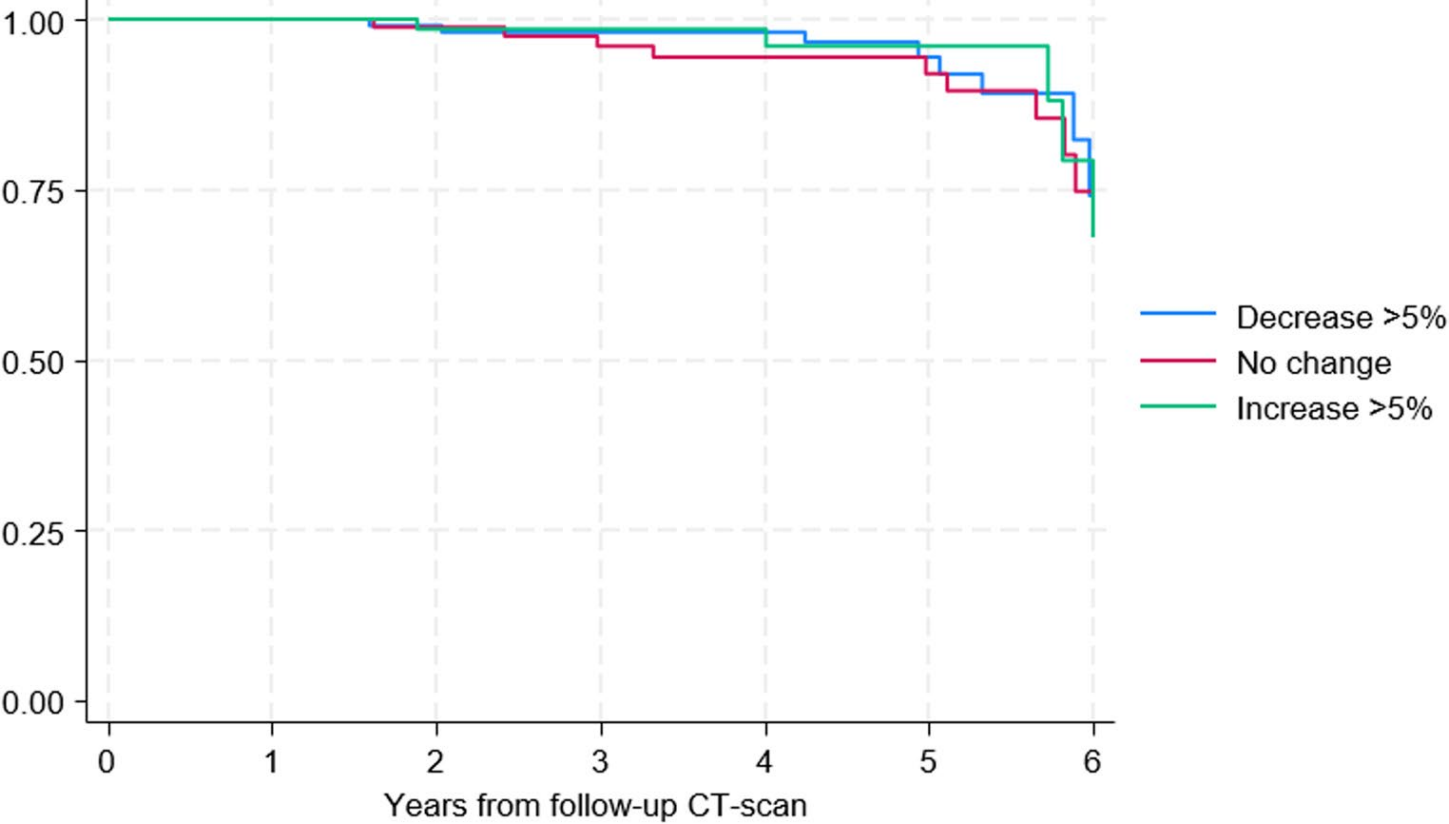
Kaplan–Meier curves for all-cause mortality by changes in psoas muscle area

There were no apparent differences in survival curves between the three groups (decrease >5 %, no change, increase >5 %) over the follow-up period (Fig. 1). These findings were supported by Cox proportional hazards regression analysis (Table 5). Using patients with no change in the psoas muscle area as the reference group, neither a >5 % decrease nor a >5% increase in psoas muscle area was associated with a statistically significant difference in mortality risk.

**Table 5:** Cox proportional hazards regression analysis of all-cause mortality by changes in psoas muscle area

Cox proportional hazard regression	Hazard Ratio (HR)	95% Confidence Interval	*P*-value
Decrease > 5%	0.75	0.29–1.90	0.543
Increase > 5%	0.70	0.24–2.06	0.522

## Discussion

In this retrospective cohort study of 272 patients undergoing surgery for CRC, we assessed longitudinal changes in psoas muscle area over a three-year postoperative period. Overall, 39% (*n* = 106) of patients experienced a > 5% decrease in psoas muscle area, 33% (*n* = 90) showed no changes and 28% (*n* = 76) had a > 5% increase. Our analysis revealed that these longitudinal changes were associated with both UICC tumor stage and treatment with adjuvant chemotherapy. Among patients who did not receive adjuvant chemotherapy, age was associated with psoas muscle area changes, whereas this association was not observed among patients who received adjuvant chemotherapy. Notably, longitudinal changes in psoas muscle area were not associated with all-cause mortality.

These findings provide important insight into the long-term dynamics of skeletal muscle in CRC survivors and their potential clinical implications. Progressive skeletal muscle loss has been linked to higher rates of postoperative complications and slower recovery [17–19] Moreover, previous studies have consistently reported that muscle loss – both preoperative and postoperative – is a strong predictor of poor overall and disease-free survival in patients with non-metastatic CRC [4, 16–19]. In contrast, our study did not observe an association between longitudinal changes in psoas muscle area and mortality. This discrepancy may be explained by several factors, including cohort characteristics, sample size, timing of muscle measurements, follow-up duration and the exclusion of patients who died or experienced recurrence within three years. By focusing on long-term survivors, our cohort was enriched for healthier patients, potentially attenuating associations between muscle loss and mortality.

In the present cohort, advanced tumor stage and treatment with adjuvant chemotherapy were associated with an increase in psoas muscle area over time. This observation contrasts with the prevailing literature, which often links chemotherapy to substantial skeletal muscle depletion, or chemotherapy-induced sarcopenia [12]. Several mechanisms contribute to this process, including systemic inflammation, metabolic dysregulation, and direct toxic effects on muscle tissue. Chemotherapy induces a pro-inflammatory state characterized by elevated levels of cytokines such as IL-6 and TNF-α, which promote proteolysis and impair muscle protein synthesis [22–24]. Prior studies have shown that skeletal muscle loss during systemic therapy is associated with poorer treatment response, progression-free survival, and overall survival in metastatic and non-metastatic CRC [19, 20].

The discrepancy between our findings and the existing literature likely reflects selection bias and cohort characteristics. In Denmark, patients ≥ 80 years are generally not offered adjuvant therapy, and those who receive chemotherapy are likely healthier, fitter, and better able to tolerate treatment. These patients may have been more frequently exposed to multidisciplinary oncology care, including nutritional assessment and dietary counselling during active treatment, which may have contributed to preservation or even gain of muscle mass. Furthermore, patients who died or experienced recurrence within three years were excluded, further enriching the cohort for long-term survivors. Differences in follow-up time and timing of muscle measurements may also contribute to the divergent results.

When muscle loss appears in patients with advanced disease it may reflect a combination of cancer-related metabolic alterations, systemic inflammation, and decreased physical activity, all of which contribute to catabolic processes and accelerated proteolysis [11]. The observed association between lower UICC tumor stage and greater muscle loss may again reflect that patients with more advanced disease are enrolled in structured oncological treatment trajectories and subjected to closer clinical surveillance.

Among patients who did not recieve adjuvant chemotherapy, age appeared to be associated with changes in psoas muscle area, with younger patients tending to gain muscle over time [4, 22]. However, this association was not formally assessed as a multivariable logistic regression model. These findings underscore the importance of considering patient-specific characteristics such as age when evaluating long-term muscle dynamics.

Furthermore, we observed that patients with a BMI > 25 exhibited > 20% loss of psoas muscle area over the three-year follow-up period. Although BMI measurements at the time of the three-year follow-up imaging were not available, the persistence of an elevated BMI would suggest a body composition phenotype consistent with sarcopenic obesity. This phenotype has been well documented in the literature and is associated with adverse oncological outcomes, including lower overall survival and poorer quality of life [25].

Muscle depletion is a clinically relevant issue in patients with CRC, as it has been associated with higher postoperative complication rates [20, 21]. Although we did not observe an association between psoas muscle area changes and mortality, this does not preclude the importance of muscle mass in postoperative recovery and quality of life. Surgical stress can exacerbate muscle loss through catabolic mechanisms, including inflammatory and hormonal responses that accelerate proteolysis and impair protein synthesis. In frail or elderly patients, these effects may be particularly pronounced, highlighting the need for targeted nutritional and physical rehabilitation strategies to counteract muscle loss and support recovery. While our retrospective design did not allow for assessment of the surgical stress response, future prospective studies should explore this component and its potential influence on long-term muscle changes.

Monitoring skeletal muscle mass has been proposed as a tool for identifying high-risk patients and guiding interventions. Our findings suggest that, in this cohort, longitudinal changes in the psoas muscle area were not associated with mortality and preoperative baseline characteristics did not predict changes in patients receiving adjuvant chemotherapy. Therefore, routine monitoring of psoas muscle area for prognostic purposes may not be warranted in all patients. Nevertheless, all patients with cancer are inherently at risk of malnutrition and muscle loss. Early identification and intervention, including nutritional assessment, exercise and multimodal rehabilitation remain critical, particularly for high-risk subgroups such as patients with stage III disease, who comprised a considerable portion of our cohort. While psoas muscle monitoring may represent a more advanced or specialized approach, consistent and systematic malnutrition screening should be advocated for all patients as a first-line strategy to improve functional and clinical outcomes.

A key strength of this study, and in contrast to most studies within this field, we assessed changes in muscle area over an extended period, with follow-up measurements conducted more than two years after the completion of adjuvant chemotherapy. Prior studies typically measure muscle area at shorter intervals, often focusing on immediate pre- and postoperative periods [26–30]. What differentiates this study is our focus on the long-term, three-year postoperative period in patients with CRC, providing a longitudinal approach to muscle changes and survival. This longer follow-up period allowed us to observe how muscle changes evolve and their potential impact over a more clinically relevant timespan. This extended follow-up may also explain why our findings contrast with existing literature, as shorter follow-up periods in previous studies might have missed long-term muscle changes. Furthermore, our use of validated CT-based measurement methods, specifically at the L3 vertebra, aligns with widely accepted protocols, thereby enhancing the reliability of our findings [31].

There are several limitations to consider in the interpretation of our study. The retrospective nature of our analysis may introduce selection bias. Additionally, patients without a three-year follow-up CT scan were excluded, which introduces survival bias. It is likely that patients with substantial decreases in psoas muscle area who died within the first year were not captured, potentially underestimating the association between muscle loss and adverse outcomes.

While we identified associations between muscle area changes and clinical outcomes, causality cannot be established due to the observational design. The study would benefit from further exploration of causal mechanisms, ideally through prospective studies and randomized trials examining targeted interventions for muscle preservation in patients with CRC. Moreover, our method of assessing muscle change using psoas muscle area at the L3 level does not fully capture whole-body skeletal muscle alterations. Future studies incorporating imaging modalities, such as total-body CT-based muscle quantification or functional assessments, may provide a broader and more comprehensive understanding of muscle dynamics in CRC patients.

In conclusion, this study revealed that UICC tumor stage and treatment with adjuvant chemotherapy were associated with longitudinal changes in psoas muscle area among patients undergoing surgery for CRC. Among patients not receiving adjuvant chemotherapy, age was associated with muscle area changes. In those receiving adjuvant chemotherapy, baseline preoperative characteristics, including age, did not uniformly predict muscle change. Importantly, alterations in psoas muscle area were not associated with mortality. These findings underscore the multifactorial nature of muscle mass dynamics in cancer survivorship, reflecting the influence of both oncologic and patient-specific factors. Future research should aim to identify patient subgroups most likely to benefit from targeted interventions to preserve muscle mass and examine whether such strategies impact outcomes beyond survival, including functional status and quality of life.

## Supplementary Information

Below is the link to the electronic supplementary material.

Supplementary Material 1

## Data Availability

The patient data that supports the findings of this study are not publicly available due to privacy and ethical restrictions but may be available from the corresponding author on reasonable request.
