# Supplementary material for: Changes in psoas muscle area in patients with colorectal cancer three years after surgery: a longitudinal approach
Source: Langenbecks Arch Surg. 2026 Mar 12;411(1):115. doi: 10.1007/s00423-026-04002-9 (PMC13056741; doi:10.1007/s00423-026-04002-9)
Supplement: Supplementary file 1 — Supplementary Material 1 [file 423_2026_4002_MOESM1_ESM.docx]

# **Changes in Psoas Muscle area in Patients with Colorectal Cancer Three Years after Surgery: A Longitudinal Approach**

*Authors:*

*Helin Yikilmaz Pardes, MD, PhD student^1,2^

ORCID-ID: 0000-0002-4780-751X

*Mona Sadek Ali, MSc, PhD^3^

ORCID-ID: 0000-0003-1152-3256

Troels Gammeltoft Dolin, MD, PhD^4^

ORCID-ID: 0000-0001-6346-3131

Casper Simonsen, MSc, PhD^8^

ORCID-ID: 0000-0003-2365-9978

Lykke Sylow, MSc, PhD

ORCID-ID: 0000-0003-0905-5932

Jakob Lykke, MD, PhD^2^

ORCID-ID: 0000-0001-9311-9964

Jacob Rosenberg, MD, DMSc^2^

ORCID-ID: 0000-0002-0063-1086

*Tinne Laurberg, MD, PhD, DMSc^6,7^

ORCID-ID: 0000-0002-7555-2665

*Louise Lang Lehrskov, MD, PhD^5,8^
ORCID-ID: ​0000-0002-1947-4252

* Marks shared authorship

*Affiliations:*
1. Department of Surgery, Zealand University Hospital, University of Copenhagen, Køge, Denmark

2. Department of Surgery, Copenhagen University Hospital – Herlev and Gentofte, Herlev, Denmark

3. Department of Biomedical Sciences, Faculty of Health and Medical Sciences, University of Copenhagen, Copenhagen, Denmark

4. Department of Medicine, Copenhagen University Hospital – Herlev and Gentofte, Herlev, Denmark

5. Department of Oncology, Copenhagen University Hospital – Herlev and Gentofte, Herlev, Denmark

6. Department of Pathology, Aarhus University Hospital – Aarhus, Denmark

7. Steno Diabetes Center Aarhus, Aarhus University Hospital, Aarhus, Denmark

8.Centre for Physical Activity Research, Copenhagen University Hospital – Rigshospitalet, Copenhagen, Denmark

*Corresponding author:*

Helin Yikilmaz Pardes
Department of Surgery, Zealand University Hospital, University of Copenhagen, Lykkebækvej 1, 4600 Køge, Denmark

Telephone: (+45): 28 91 34 73
Email: heyi@regionsjaelland.dk

*Keywords*

Colorectal cancer • Cancer surgery • Postoperative muscle loss • Psoas muscle area

*Supplementary table 1: Baseline demographics and changes in psoas muscle area with expanded categorization:*

|  | **Decrease >20%** | **Decrease 10-20%** | **Decrease 5-10%** | **Decrease 0-5%** | **Increase 0-5%** | **Increase 5-10%** | **Increase 10-20%** | **Increase >20%** | **Total** | **P-value** |
| --- | --- | --- | --- | --- | --- | --- | --- | --- | --- | --- |
| *n* (%) | 32 (11.8) | 46 (16.9) | 28 (10.3) | 38 (14.0) | 52 (19.1) | 27 (9.9) | 27 (9.9) | 22 (8.1) | 272 (100.0) |  |
| Sex, *n* (%)  Male  Female | 14 (43.8)  18 (56.2) | 23 (50.0)  23 (50.0) | 10 (35.7)  18 (64.3) | 16 (42.1)  22 (57.9) | 29 (55.8)  23 (44.2) | 15 (55.6)  12 (44.4) | 13 (48.1)  14 (51.9) | 10 (45.5)  12 (54.5) | 130 (47.8)  142 (52.2) | 0.73 |
| Age, year, mean (sd) | 69.1 (7.0) | 65.9 (9.9) | 65.9 (8.2) | 67.3 (9.2) | 67.5 (7.5) | 67.4 (9.3) | 64.2 (11.2) | 64.6 (10.7) | 66.7 (9.1) | 0.44 |
| BMI kg/m^2^, mean (sd) | 26.0 (4.9) | 26.2 (4.0) | 26.3 (4.4) | 25.5 (4.9) | 25.7 (5.4) | 26.4 (4.3) | 25.5 (3.0) | 25.1 (3.2) | 25.8 (4.4) | 0.96 |
| Baseline mean of total psoas area (sd) | 16.4 (5.1) | 16.6 (4.8) | 15.2 (4.2) | 14.9 (5.9) | 15.7 (4.6) | 14.7 (4.7) | 14.9 (5.2) | 12.8 (3.9) | 15.4 (4.9) | 0.11 |
| Follow-up mean of total psoas area (sd) | 11.6 (4.3) | 14.1 (4.0) | 14.1 (3.9) | 14.6 (5.8) | 16.1 (4.8) | 15.8 (5.1) | 17.0 (5.8) | 17.8 (5.0) | 15.0 (5.1) | 0.00 |
| CRP, mg/L, median (IQR) | 3.0 (3.0–7.4) | 2.3 (1.0-3.9) | 3.0 (3.0–4.6) | 3.9 (3.0-7.0) | 3.0 (1.9-4.4) | 3.4 (2.2-7.0) | 4.4 (2.4-15) | 3.0 (3.0–7.8) | 3.0 (1.9–4.7) | 0.11 |
| Actively smoking, *n (*%) | 7 (21.9) | 4 (8.7) | 2 (7.1) | 7 (18.4) | 5 (9.8) | 4 (14.8) | 3 (11.5) | 4 (19.0) | 36 (13.4) | 0.55 |
| Alcohol intake (>= 7 units pr week), *n* (%) | 9 (29.0) | 14 (30.4) | 8 (28.6) | 11 (29.7) | 18 (36.0) | 7 (25.9) | 4 (14.8) | 3 (14.3) | 74 (27.7) | 0.52 |
| ASA score, *n* (%)  1  2  3 | 14 (43.8)  15 (46.9)  3 (9.4) | 19 (42.2)  22 (48.9)  4 (8.9) | 11 (39.3)  16 (57.1)  1 (3.6) | 16 (43.2)  20 (54.1)  1 (2.7) | 23 (44.2)  26 (50.0)  3 (5.8) | 10 (37.0)  13 (48.1)  4 (14.8) | 13 (48.1)  12 (44.4)  2 (7.4) | 8 (36.4)  13 (59.1)  1 (4.5) | 114 (42.2)  137 (50.7)  19 (7.0) | 0.96 |
| UICC stage, *n* (%)  I  II  III | 17 (53.1)  9 (28.1)  6 (18.8) | 21 (45.7)  14 (30.4)  11 (23.9) | 13 (46.4)  10 (35.7)  5 (17.9) | 15 (39.5)  11 (28.9)  12 (31.6) | 14 (26.9)  21 (40.4)  17 (32.7) | 8 (29.6)  11 (40.7)  8 (29.6) | 7 (25.9)  5 (18.5)  15 (55.6) | 6 (27.3)  11 (50.0)  5 (22.7) | 101 (37.1)  92 (33.8)  79 (29.0) | 0.07 |
| Type of surgery, *n* (%)  Hemicolectomy  Sigmoidectomy  Rectum resection (Total mesorectal excision) | 10 (31.2)  7 (21.9)  15 (46.9) | 15 (32.6)  16 (34.8)  15 (32.6) | 9 (32.1)  10 (35.7)  9 (32.1) | 18 (47.4)  7 (18.4)  13 (34.2) | 22 (42.3)  17 (32.7)  13 (25.0) | 11 (40.7)  9 (33.3)  7 (25.9) | 12 (44.4)  9 (33.3)  6 (22.2) | 8 (36.4)  8 (36.4)  6 (27.3) | 105 (38.6)  83 (30.5)  84 (30.9) | 0.73 |
| Adjuvant chemotherapy, *n* (%) | 7 (21.9) | 11 (23.9) | 5 (17.9) | 11 (28.9) | 21 (40.4) | 13 (48.1) | 15 (55.6) | 8 (36.4) | 91 (33.5) | *0.02** |

*Values are given as absolute numbers (percentages) in parentheses unless otherwise is stated.*

*Statistical significance is denoted as follows: p<0.05 (*)*

*Abbreviations: BMI: Body Mass Index, CRP: C-reactive protein, ASA: American Society of Anesthesiology score, UICC: Union for International Cancer Control*
